# On the Security of a Novel Probabilistic Signature Based on Bilinear Square Diffie-Hellman Problem and Its Extension

**DOI:** 10.1155/2014/345686

**Published:** 2014-06-12

**Authors:** Zhenguo Zhao, Wenbo Shi

**Affiliations:** ^1^School of Water Conservancy, North China University of Water Resources and Electric Power, Zhengzhou 450045, China; ^2^Department of Electronic Engineering, Northeastern University at Qinhuangdao, Qinhuangdao 066004, China

## Abstract

Probabilistic signature scheme has been widely used in modern electronic commerce since it could provide integrity, authenticity, and nonrepudiation. Recently, Wu and Lin proposed a novel probabilistic signature (PS) scheme using the bilinear square Diffie-Hellman (BSDH) problem. They also extended it to a universal designated verifier signature (UDVS) scheme. In this paper, we analyze the security of Wu et al.'s PS scheme and UDVS scheme. Through concrete attacks, we demonstrate both of their schemes are not unforgeable. The security analysis shows that their schemes are not suitable for practical applications.

## 1. Introduction

Signature scheme is an important modern cryptographic mechanism of the public key cryptosystem. In the signature scheme, the signer uses his private key to sign a message and generate a signature, which could be verified by other users using the signer's public key. The signature could provide integrity, authenticity, and nonrepudiation; then it could be used in modern electronic commerce [1–5].

The undeniable signature (US) scheme is a variation of the signature scheme, which was first introduced by Chaum and van Antwerpen [6]. In the US scheme, the verifier should get the signer's cooperation to finish the verification. In order to remove the complicated cooperation between the signer and the verifier, Jakobsson et al. [7] introduced the concept of the designated verifier signature (DVS) scheme and proposed a concrete DVS scheme. However, Wang [8] found that there is serious security vulnerability in Jakobsson et al.'s scheme. Later, Steinfeld et al. [9, 10] introduced the concept of the universal designated verifier signature (UDVS) scheme to generate the concept of the DVS scheme. In the UDVS scheme, the signer could generate a signature and only the designated verifier could verify the signature using his private key.

Later, Zhang et al. [11] used Diffie-Hellman problem to construct a UDVS scheme and demonstrated that their scheme is provably secure in the standard model. Unfortunately, Cheon [12] found that Zhang et al.'s scheme had a security flaw. To enhance security, Huang et al. [13] presented a new UDVS scheme using the gap bilinear Diffie-Hellman problem. In order to satisfy applications in identity-based systems, Chen et al. [14] proposed the first identity-based UDVS scheme. In order to improve efficiency, Wu and Lin [15] proposed a probabilistic signature (PS) scheme using the bilinear square Diffie-Hellman (BSDH) problem. Then, they extended this PS scheme to a UDVS scheme. They also demonstrated that both of their schemes are provably secure in the random oracle. In this paper, we analyze the security of both Wu and Lin's PS scheme and UDVS scheme. Through concrete attacks, we show that neither of their schemes is unforgeable. We will also propose efficient countermeasures to withstand those attacks.

The organization of the paper is sketched as follows.  Section 2 gives a brief review of Wu et al.'s PS scheme and UDVS scheme.  Section 3 presents our attacks against Wu et al.'s PS scheme and UDVS scheme.  Section 4 presents our countermeasures to withstand the proposed attacks. At last, Section 5 presents some conclusion of the paper.

## 2. Review of Wu and Lin's Schemes

In this section, we will give the details of Wu et al.'s PS scheme and UDVS scheme.

### 2.1. Review of Wu and Lin's PS Scheme

There are two participants in Wu and Lin's PS scheme, that is, a signer and a verifier, where the signer generates a publicly verifiable signature (PV-signature) using his private key and the verifier could verify the validity of the PV-signature using the signer's public key. There are three algorithms in Wu and Lin's PS scheme, that is,* Setup*,* PV-Signature-Generation*, and* PV-Signature-Verification*.

*Setup*. Taking a security parameter *k* as input, the system authority (SA) runs the following steps to generate system parameters. Besides, the user *U*_*i*_ registers his public key.

1. SA chooses a random number *q* and selects two multiplicative groups (*G*_1_, ×) and (*G*_2_, ×) with the same order *q*, where the bit length of *q* is *k*.
2. SA chooses a generator *P* of the group (*G*_1_, ×) and a bilinear pairing *e* : *G*_1_ × *G*_1_ → *G*_2_.
3. SA chooses two secure hash functions *h*_1_ and *h*_2_, where *h*_1_ : *G*_1_ → *G*_1_ and *h*_2_ : {0,1}* × *G*_1_ → *Z*_*q*_.
4. SA publishes the system parameters *params* = {*G*_1_, *G*_2_, *q*, *P*, *e*, *h*_1_, *h*_2_}.
5. *U*_*i*_ chooses a random number *x*_*i*_ ∈ *Z*_*q*_ as his private key and registers his public key *Y*_*i*_ = *P* ^*x*_*i*_^.

*PV-Signature-Generation*. Upon receiving the message *m*, the signer *U*_*s*_ runs the following steps to generate a PV-signature Ω.

1. *U*_*s*_ chooses a random number *r* ∈ *Z*_*q*_ and computes *R* = *P* ^*r*^, *T* = *h*_1_(*R*)^*x*_*s*_^, and *ρ* = *h*_2_(*m*, *R*)*x*_*s*_^2^ − *r*mod⁡*q*.
2. *U*_*s*_ outputs Ω = (*R*, *T*, *ρ*) as the PV-signature of the message *m*.

*PV-Signature-Verification*. Upon receiving the message *m*, the PV-signature Ω, and the signer's public key *Y*_*s*_, the verifier *U*_*v*_ runs the following steps to verify the validity of the PV-signature.

1. *U*_*v*_ checks whether the equation *e*(*P* ^*ρ*^ *R*, *h*_1_(*R*)) = *e*(*Y*_*s*_, *T*)^*h*_2_(*m*,*R*)^ holds.
2. If the equation holds, *U*_*v*_ confirms the PV-signature is valid; otherwise, *U*_*v*_ confirms that the PV-signature is not valid.

### 2.2. Review of Wu and Lin's UDVS Scheme

There are two participants in Wu and Lin's UDVS scheme, that is, a signer and a verifier, where the signer generates a designated verifiable signature (DV-signature) using his private key and only the designated verifier could verify the validity of the DV-signature using the signer's public key. There are five algorithms in Wu and Lin's UDVS scheme, that is,* Setup*,* PV-Signature-Generation*,* PV-Signature-Verification*,* DV-Signature-Generation*, and* DV-Signature-Verification*. Because the first three algorithms are the same as those in PS scheme, only the last two algorithms will be described in detail.

*DV-Signature-Generation*. Upon receiving a message *m* and the designated verifier *U*_*v*_'s public key *Y*_*v*_, the signer *U*_*s*_ runs the following steps to generate a DV-signature Ω.

1. *U*_*s*_ chooses a random number *r* ∈ *Z*_*q*_ and computes *R* = *P* ^*r*^, *T* = *h*_1_(*R*)^*x*_*s*_^, *ρ* = *h*_2_(*m*, *R*)*x*_*s*_^2^ − *r*mod⁡*q*, and *W* = *Y*_*v*_^*ρ*^.
2. *U*_*s*_ outputs Ω = (*R*, *T*, *W*) as the DV-signature of the message *m*.

*DV-Signature-Verification*. Upon receiving a message *m*, the DV-signature Ω, and the signer's public key *Y*_*s*_, the designated verifier *U*_*v*_ runs the following steps to verify the validity of the DV-signature.

1. *U*_*v*_ checks whether the equation *e*(*W* ^*x*_*v*_^−1^^ *R*, *h*_1_(*R*)) = *e*(*Y*_*s*_, *T*)^*h*_2_(*m*,*R*)^ holds.
2. If the equation holds, *U*_*v*_ confirms the DV-signature is valid; otherwise, *U*_*v*_ confirms that the PV-signature is not valid.

## 3. Security Analysis of Wu and Lin's Schemes

In this section, we will give the security analysis of Wu et al.'s PS scheme and UDVS scheme.

### 3.1. Security Analysis of Wu and Lin's PS Scheme

Wu and Lin claimed that their PS scheme was unforgeable against various attacks. Through concrete attack, we will show that an adversary without the signer *U*_*s*_'s private key could forge a legal PV-signature of any message. Given a message *m*, the adversary *A* could forge a legal PV-signature through the following steps.

1. *A* generates a random number *ρ* ∈ *Z*_*q*_ and computes *R* = *Y*_*s*_ *P* ^−*ρ*^ and *T* = *h*_1_(*R*)^*h*_2_(*m*,*R*)^−1^mod⁡*q*^.
2. *A* outputs Ω = (*R*, *T*, *ρ*) as the PV-signature of the message *m*.

Since *R* = *Y*_*s*_ *P* ^−*ρ*^ and *T* = *h*_1_(*R*)^*h*_2_(*m*,*R*)^−1^mod⁡*q*^, we could get

(1) e(PρR,h1(R))  =e(PρYsP−ρ,h1(R))  =e(Ys,h1(R)),e(Ys,T)h2(m,R)  =e(Ys,h1(R)h2(m,R)−1mod⁡q)h2(m,R)  =e(Ys,h1(R))h2(m,R)·h2(m,R)−1mod⁡q  =e(Ys,h1(R)).

From (1), we know that the equation *e*(*P* ^*ρ*^ *R*, *h*_1_(*R*)) = *e*(*Y*_*s*_, *T*)^*h*_2_(*m*,*R*)^ holds. Then, the PV-signature generated by the adversary could pass the verifier's check. Therefore, the adversary could forge a legal PV-signature.

### 3.2. Security Analysis of Wu and Lin's UDVS Scheme

Wu and Lin claimed that their UDVS scheme was unforgeable against various attacks. Through concrete attack, we will show that an adversary without the signer *U*_*s*_'s private key could forge a legal DV-signature of any message. Given a message *m* and the designated verifier *U*_*v*_'s public key *Y*_*v*_, the adversary *A* could forge a legal DV-signature through the following steps.

1. *A* generates a random number *ρ* ∈ *Z*_*q*_ and computes *R* = *Y*_*s*_ *P* ^−*ρ*^, *T* = *h*_1_(*R*)^*h*_2_(*m*,*R*)^−1^mod⁡*q*^ and *W* = *Y*_*v*_^*ρ*^.
2. *A* outputs Ω = (*R*, *T*, *ρ*) as the DV-signature of the message *m*.

Since *R* = *Y*_*s*_ *P* ^−*ρ*^, *T* = *h*_1_(*R*)^*h*_2_(*m*,*R*)^−1^mod⁡*q*^, *W* = *Y*_*v*_^*ρ*^, and *Y*_*v*_ = *P* ^*x*_*v*_^, we could get

(2) e(Wxv−1R,h1(R))  =e((Yvρ)xv−1YsP−ρ,h1(R))  =e(((Pxv)ρ)xv−1YsP−ρ,h1(R))  =e(Pxvρxv−1YsP−ρ,h1(R))  =e(PρYsP−ρ,h1(R))  =e(Ys,h1(R)),e(Ys,T)h2(m,R)  =e(Ys,h1(R)h2(m,R)−1mod⁡q)h2(m,R)  =e(Ys,h1(R))h2(m,R)·h2(m,R)−1mod⁡q  =e(Ys,h1(R)).

From (2), we know that the equation *e*(*W* ^*x*_*v*_^−1^^ *R*, *h*_1_(*R*)) = *e*(*Y*_*s*_, *T*)^*h*_2_(*m*,*R*)^ holds. Then, the DV-signature generated by the adversary could pass the verifier's verification. Therefore, the adversary could forge a legal DV-signature.

## 4. Countermeasures

### 4.1. Countermeasure for Wu and Lin's PS Scheme

From the details of Wu and Lin's PS scheme, we know that the value *T* has no relation with the value of *ρ*. Then the adversary could choose the value *T* freely to remove the relation between *R* and *ρ*. To withstand the attack described in Section 3.1, we just need to modify Wu and Lin's PS scheme slightly.

*DV-Signature-Generation*. Upon receiving a message *m*, the signer *U*_*s*_ runs the following steps to generate a PV-signature Ω.

1. *U*_*s*_ chooses a random number *r* ∈ *Z*_*q*_ and computes *R* = *P* ^*r*^, *T* = *h*_1_(*R*)^*x*_*s*_^, and *ρ* = *h*_2_(*m*, *R*, *T*)*x*_*s*_^2^ − *r*mod⁡*q*.
2. *U*_*s*_ outputs Ω = (*R*, *T*, *ρ*) as the PV-signature of the message *m*.

*DV-Signature-Verification*. Upon receiving a message *m*, the PV-signature Ω, and the signer's public key *Y*_*s*_, the verifier *U*_*v*_ runs the following steps to verify the validity of the PV-signature.

1. *U*_*v*_ checks whether the equation *e*(*P* ^*ρ*^ *R*, *h*_1_(*R*)) = *e*(*Y*_*s*_, *T*)^*h*_2_(*m*,*R*,*T*)^ holds.
2. If the equation holds, *U*_*v*_ confirms the PV-signature is valid; otherwise, *U*_*v*_ confirms that the PV-signature is not valid.

After the modification, the adversary *A* could generate a random number *ρ* ∈ *Z*_*q*_ and compute *R* = *Y*_*s*_ *P* ^−*ρ*^, *T* = *h*_1_(*R*)^*h*_2_(*m*,*R*)^−1^mod⁡*q*^. However, the equation *e*(*P* ^*ρ*^ *R*, *h*_1_(*R*)) = *e*(*Y*_*s*_, *T*)^*h*_2_(*m*,*R*,*T*)^ never holds since the adversary cannot use *h*_2_(*m*, *R*)^−1^mod⁡*q* to remove the function *h*_2_(*m*, *R*, *T*)^−1^mod⁡*q*. Then, the modified scheme is secure against the attack described in Section 3.1.

### 4.2. Countermeasure for Wu and Lin's UDVS Scheme

From the details of Wu and Lin's UDVS scheme, we know that the value *T* has no relation to the value of *W*. Then the adversary could choose the value *T* freely to remove the relation between *R* and *W*. To withstand the attack described in Section 3.2, we just need to modify Wu and Lin's UDVS scheme slightly.

*DV-Signature-Generation*. Upon receiving a message *m* and the designated verifier *U*_*v*_'s public key *Y*_*v*_, the signer *U*_*s*_ runs the following steps to generate a DV-signature Ω.

1. *U*_*s*_ chooses a random number *r* ∈ *Z*_*q*_ and computes *R* = *P* ^*r*^, *T* = *h*_1_(*R*)^*x*_*s*_^, *ρ* = *h*_2_(*m*, *R*, *T*)*x*_*s*_^2^ − *r*mod⁡*q*, and *W* = *Y*_*v*_^*ρ*^.
2. *U*_*s*_ outputs Ω = (*R*, *T*, *W*) as the DV-signature of the message *m*.

*DV-Signature-Verification*. Upon receiving a message *m*, the DV-signature Ω, and the signer's public key *Y*_*s*_, the designated verifier *U*_*v*_ runs the following steps to verify the validity of the DV-signature.

1. *U*_*v*_ checks whether the equation *e*(*W* ^*x*_*v*_^−1^^ *R*, *h*_1_(*R*)) = *e*(*Y*_*s*_, *T*)^*h*_2_(*m*,*R*,*T*)^ holds.
2. If the equation holds, *U*_*v*_ confirms the DV-signature is valid; otherwise, *U*_*v*_ confirms that the PV-signature is not valid.

After the modification, the adversary *A* could generate a random number *ρ* ∈ *Z*_*q*_ and compute *R* = *Y*_*s*_ *P* ^−*ρ*^, *T* = *h*_1_(*R*)^*h*_2_(*m*,*R*)^−1^mod⁡*q*^ and *W* = *Y*_*v*_^*ρ*^. However, the equation *e*(*W* ^*x*_*v*_^−1^^ *R*, *h*_1_(*R*)) = *e*(*Y*_*s*_, *T*)^*h*_2_(*m*,*R*,*T*)^ never holds since the adversary cannot use *h*_2_(*m*, *R*)^−1^mod⁡*q* to remove the function *h*_2_(*m*, *R*, *T*)^−1^mod⁡*q*. Then, the modified scheme is secure against the attack described in Section 3.2.

## 5. Conclusion

Recently, Wu and Lin proposed a PS scheme using the bilinear square Diffie-Hellman problem and extended it to a UDVS scheme. They also demonstrated that their scheme is provably secure in the random oracle. Through concrete attacks, we demonstrate that neither of their schemes is unforgeable against common adversary. To improve security, we also propose efficient countermeasures to withstand the proposed attacks.
